# Montane evergreen forest deforestation for banana plantations decreased soil organic carbon and total nitrogen stores to alarming levels

**DOI:** 10.1186/s13021-024-00278-w

**Published:** 2024-08-29

**Authors:** Tarquinio Mateus Magalhães, Edna Rita Bernardo Cossa, Hunilcia Esperança Nhanombe, Amélia David Muchanga Mugabe

**Affiliations:** Departamento de Engenharia Florestal, Universidade Eduardo Mondlane, Av. Julius Nyerere no. 3453, Campus Universitário Principal, Edifício no. 1, 257 Maputo, Moçambique, South Africa

**Keywords:** Agriculture, Eastern Africa, Biomass, Land-use change, Soil nutrients

## Abstract

Forest conversion to agricultural land has been shown to deplete soil organic carbon (SOC) and soil total nitrogen (STN) stocks. However, research on how soil properties respond to forest conversion to shifting cultivation has produced conflicting results. The conflicting findings suggest that the agricultural system may influence the response of SOC and STN to forest conversion to agriculture, depending on the presence of vegetative cover throughout the year. Due to the unique characteristics of montane evergreen forests (MEF) and banana plantations (BP), SOC and STN response to MEF conversion to BP may differ from existing models. Nevertheless, research on how soil properties are affected by MEF conversion to BP is scarce globally. In order to fill this research gap, the goal of this study was to evaluate how much deforestation for BP affects SOC, STN, and soil quality by analysing these soil parameters in MEF and BP fields down to 1-m depth, using standard profile-based procedures. Contrary to the specified hypothesis that SOC and STN losses would be restricted to the upper 20-cm soil layer, SOC losses were extended to the 40-cm depth layer and STN losses to the 60-cm depth layer. The soils lost 18.56 Mg ha ^– 1^ (37%) of SOC from the upper 20 cm and 33.15 Mg ha ^– 1^ (37%) from the upper 40 cm, following MEF conversion to BP. In terms of STN, the upper 20, 40, and 60 cm lost 2.98 (43%), 6.62 (47%), and 8.30 Mg ha ^– 1^ (44%), respectively. Following MEF conversion to BP, the SOC stratification ratio decreased by 49%, implying a decline in soil quality. Massive exportation of nutrients, reduced C inputs due to complete removal of the arboreal component and crop residues, the erodibility of the soils on the study area’s steep hillslopes, and the potential for banana plantations to increase throughfall kinetic energy, and splash erosion through canopy dripping are thought to be the leading causes of SOC and STN losses. More research is needed to identify the extent to which each cause influences SOC and STN losses.

## Introduction

Montane evergreen forests (MEF) account for the majority of the remaining evergreen old-growth forest cover in eastern Africa [1]. They are an important component of several of the world’s biodiversity hotspots because they represent an extraordinarily species-rich system that is becoming increasingly endangered owing to human intervention [2].

With estimated tree carbon (C) reserves of 149 Mg ha ^– 1^, MEF of eastern Africa’s mountains have more concentrated C storage than the Amazon and other lowland African rainforests [1]. However, because 0.8 million hectares of old-growth eastern African montane forest have been lost since 2000 [1], this C store is in danger of becoming a C source rather than a sink. According to Hamunyela et al. [3], in eastern Africa, the vulnerability of MEF to human-induced disturbances is aggravated in areas highly suitable for agricultural production. MEF are primarily deforested for banana and maize croplands in these places [3]. Agriculture expansion is the primary source of deforestation, accounting for 90% of global deforestation and 75% of deforestation in eastern Africa [4].

MEF span around 182,131 hectares in Mozambique, accounting for 0.23% of the country’s total area and 0.56% of the wooded area [5, 6]. This forest type is found in the hilly areas of the following districts [5]: Gurùé (Namúli Massif), Milange (Mount Chiperone), Manica (Mount Vumba), Gorongosa (Mount Gorongosa), Mueda (Mueda Plateau), and Sussundenga (the Chimanimani Mountain Range). The Moribane Forest Reserve (MFR), the study area, is part of the Chimanimani Mountains, and has one of the montane evergreen forests that has suffered the most from deforestation for agriculture since the early 2000s, particularly for BP. Banana is the most important fruit and one of the most important crops in the world [7, 8], and the primary source of income for the Moribane and nearby communities.

Soil organic carbon (SOC) and soil total nitrogen (STN) are the most commonly used soil quality indicators in the literature, and they are regarded as the best indices of soil rehabilitation [9] and fertility in both managed and natural ecosystems [10, 11]. Land use and land-use change have an impact on SOC and STN stocks due to differences in organic matter input quantities [12]. Conversion of forest to agricultural land has been shown to deplete SOC and STN stocks [13–19]. However, research on how SOC and STN respond to forest conversion to shifting cultivation, particularly miombo, has produced conflicting results. While some studies [20–23] report no changes, others [24, 25] report depletion. The conflicting findings suggest that the agricultural system may influence SOC and STN responses to forest conversion to agriculture [22]. Magalhães [22] found that converting a forest to a tree-based farming system did not affect SOC levels, but converting to a treeless farming system depleted SOC stores. Similarly, especially in the study area, SOC and STN responses to MEF conversion to BP may deviate from existing models for various reasons:

1. Because MEF occurs on steep hillslopes, its soils are vulnerable to additional sources of SOC and STN loss when converted to BP, including as physical removal through leaching and erosion [26].
2. Banana plants export relatively larger amounts of nutrients through fruit harvesting [27, 28], because of their greater nutrient demand [29]. To meet their nutritional demands, plants degrade soil organic matter (SOM, the soil’s only reservoir of easily absorbed nutrients) [30], reducing SOC and STN reserves. In the research area, banana pseudostems and leaves are used to make baskets for banana transportation, resulting in no or little SOM inputs, low nutrient recycling and increased nutrient exportation. Furthermore, no fertilizer is used to restore the nutrients exported.
3. Banana canopy is known to promote throughfall erosivity and splash erosion [31–34], potentially increasing losses in SOC and STN.
4. In contrast to certain agricultural systems, where all of the plants are removed from the land with the annual harvest, leaving the soil exposed until the next cropping season, selective harvesting in BP keeps the soil covered throughout the year. Covered soils reduce SOC and STN losses by minimizing exposure to environmental conditions that increase SOM decomposition [13, 35, 36].

Despite the above-mentioned information and the abundance of studies on the consequences of forest conversion to agriculture on SOC and STN, the impacts of MEF deforestation for agriculture, particularly for BP, on SOC and STN have received little attention, both regionally and globally. To the best of our knowledge, Powers (2004) [37] is the only study on this issue worldwide. Comparable studies conducted in eastern and southern Africa have concentrated on the conversion of lowland miombo woodlands to agricultural lands, excluding BP [21–24, 38–41].

In an effort of addressing the apparent research gap that was previously mentioned, the objective of this study was to determine how much deforestation for BP affects SOC, STN, and soil quality. Because only mature bananas are collected during banana harvesting, not the entire plantation, it was predicted that SOC and STN losses would be minimal and restricted to the surface soil (20 cm depth) due to the year-round presence of vegetative cover in BP fields.

## Materials and methods

### Study area

**Fig. 1:**
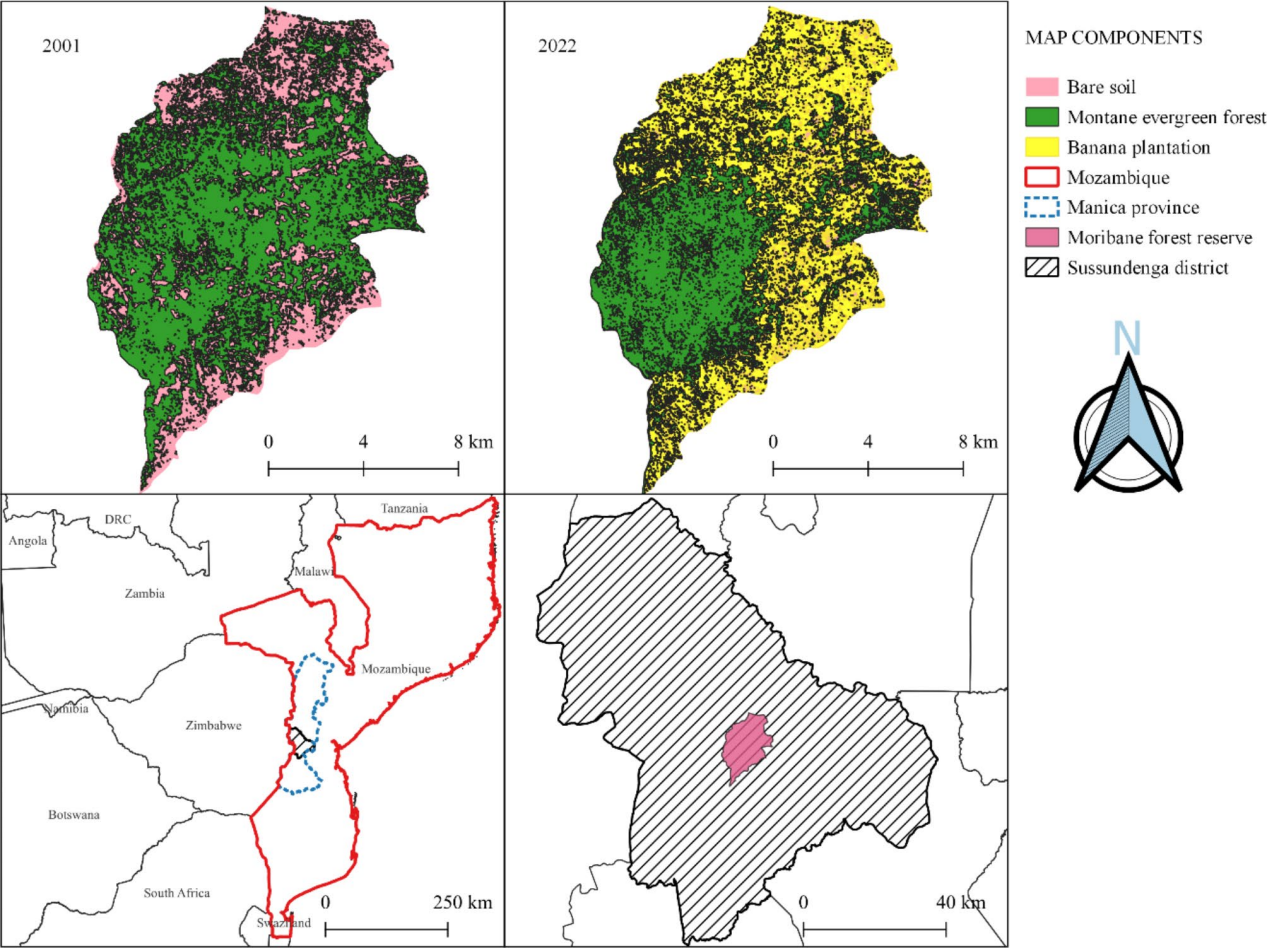
Location map of the study area and land use and land cover change map of 2001 and 2022

MFR is located between 33.28^o^ to 33.35^o^ E and − 19.84^o^ to − 19.66^o^ S. It is a 161 km2 reserve in the Sussundenga district of Mozambique’s Manica province (Fig. 1), and is part of buffer zone of the Chimanimani Transfrontier Conservation Area. The reserve’s major vegetation type is montane evergreen forest (MEF), which has gradually been replaced by banana plantations (BP) since the early 2000s, despite the government’s efforts to curb agricultural growth. Between 2001 and 2022 (Fig. 1), BP fields expanded from 0 to 7369 ha, while MEF lands declined from 10,232 to 6972 ha, representing a 32% (3260 ha) loss. Both the transformation in land cover from MEF and bare soil to BP resulted in the 7369 hectares of BP land. The bare soils were caused by forest fires in 1992, followed by a prolonged drought [42].

Agricultural preparation of soil in BP fields is done by hoe, with no mechanical agitation, culminating in no-till farming, and no fertilizers or pesticides are used. MRF is located in the mountainous region of the Chimanimani Mountain Range, which has a terrain that is plateau-mountainous, steep, with massive slopes, extensive plains, and a vast hydrographic basins [43, 44]. The average annual precipitation ranges from 1200 to 1400 mm [44], and the average annual temperature is 24 °C, while the minimum temperature can reach 9 °C during the cold season [45]. The dominant soil types of the area are Rhodic Ferralsols, Eutric Leptosols, and Albic Arenosols, covering 63, 24 and 13% of the whole study area [46], respectively. The relevant land-use and land cover (LULC) classes are forests and agriculture (mainly banana plantations).

### Sampling and computation

Diameter at breast height (DBH) was measured on trees with DBH ≥ 5 cm using a fixed area sampling method [47–49] and a stratified random sampling design. There were two strata studied: MEF and BP. Here, a landform that rises at least 300 m above its surroundings or any elevation of land mass from the plains 300 m.a.s.l. is referred to as a mountain [50, 51]. Thirty-one (31) square plots were placed at random across the strata, with 15 in the forest and 16 in the banana plantations. Each slope aspect (north- and south-facing) in the MEF stratum received 8 and 7 plots, respectively. The plots ranged in elevation from 434 to 778 m.a.s.l, with an average of 683 ± 14 m.a.s.l. Plots in the BP stratum were spread evenly in each slope aspect, with elevations ranging from 409 to 772 m.a.s.l (average = 632 ± 16 m.a.s.l.). The BP plots’ slope angles ranged from 4 to 21 degrees, whereas the MEF plots’ slope angles ranged from 6 to 23 degrees. This study focused on C changes in tree biomass, tree necromass, and soil, as well as STN changes after MEF conversion to BP. The carbon stored in banana biomass was not considered. This study did not include recent BP lands (within the last 5 years), because newly converted land-use systems can track the soil properties of the prior use. Additionally, BP established in previously bare soils was not taken into account. Because most dendrometric estimates are given in terms of horizontal per unit area, the plots were corrected for slope angle so that the horizontal projected area is 625 m^2^ (25 m × 25 m), as specified.

Aboveground tree dry mass was calculated using allometric equations (Eq. 1) developed by Lisboa et al. [42] for the research area. The global root-to-shoot ratio of 0.25 was used to calculate belowground dry mass [52]. To achieve the plot-level dry mass, the relevant tree-level dry masses were added.

1 $$\:{\widehat{\text{Y}}}_{\text{A}\text{G}\text{B}}=0.0613\times\:{\text{D}\text{B}\text{H}}^{2.7133}$$

where Ŷ_AGB_ is the estimate of aboveground tree dry mass.

Standing coarse woody debris (CWD) and fallen CWD were separated, with the former including only snags (dead trees). There were no dead or living stumps found. Snags were measured for DBH and its above- and belowground dry mass obtained similarly to living trees. This study did not take into account dead wood attached to a living tree (e.g. branches). Fallen CWD were classified into small CWD (diameter between 2.5 and 7.5 cm) and large CWD (diameter ≥ 7.5 cm). Each large CWD found in the plot area was quantified for volume using the Newton’s method [47, 49] and its scientific name recorded, whenever possible. The dry mass was calculated by dividing the volume by the appropriate basic density from the literature [53]. The dry mass of the unidentified CWD was calculated based on the average basic density of the CWD found in the relevant plot.

A 5 m × 5 m quadrat was set up in the plot’s west-top corner to measure the fresh mass of fine CWD. Fresh mass measurements of fine woody debris (FWD, diameter < 2.5 cm) and litter were taken in a 1 m × 1 m quadrat set up on the plot’s westernmost corner. In a 1 m × 1 m quadrat set up in the plot’s east-top corner, grasses and herbs were chopped and fresh-weighted. A fresh-weighted subsample of 5–10% of the fresh mass of the quadrat material was sent to the laboratory for oven-drying, and the respective dry mass was estimated by multiplying the oven-dry-to-fresh-mass ratio of the subsample by the total fresh mass.

C stock was estimated by multiplying dry mass by 0.5, assuming that C accounts for 50% of the dry mass [54–56].

A soil pit was excavated in the centre of each plot to disclose the soil profile. Undisturbed soil samples (soil cores) were taken perpendicularly to the soil profile using a 100 cm^3^ volume corer (height: 51 mm, inner diameter: 50 mm) at five previously split soil depth (D) layers (D1: 0–20 cm, D2: 20–40 cm, D3: 40–60 cm, D4: 60–80 cm, and D5: 80–100 cm). For the representativeness of the soil layer, the soil cores were obtained at the central point. The 155 soil samples were all transferred to the lab for bulk density determination.

The soil samples were powdered to pass through a 2-mm sieve, oven-dried at 105 ± 2^o^ C to constant mass, weighted, and the mass of the resultant rock fragments (RF, Ø ≥2 mm) quantified. The mass of the fine soil (M_FS_) was obtained as the difference between the total dry mass of the core soil sample (M_S_) and that of the rock fragments (M_RF_), and the mass fraction of rock fragments as the ratio between M_RF_ and M_S_. The STN and SOC concentrations of the fine soil were ascertained using the Kjeldahl and Walkley and Black techniques [57]. By dividing the M_FS_ by its volume (V_FS_), the bulk density of the fine soil (BD_FS_) was computed. V_FS_ is the difference between the volume of the corer (V_C_) and the volume of the rock fragments (V_RF_). V_RF_ was calculated as the ratio of M_RF_ to the density of the rock fragments, which is estimated to be 2.6 g cm ^– 3^ [58].

It is anticipated that the conversion of MEF to BP will be accompanied by increases in soil bulk density brought on by farming operations that compact the soil, and decreases in biomass-mediated adsorption of organic matter to the soil as a result of the removal of trees. As a result, the sampled mass and volume of soils per unit area will differ between the two land-use systems (MEF and BF). Therefore, comparisons of SOC and STN concentrations and stocks at fixed depth (FD) intervals of the two land-use systems will be prone to errors and confounded by differences in soil mass and volume [59–61].

Due to the aforementioned, the equivalent soil mass (EMS) method was utilized to calculate SOC and STN stocks using the bulk density, SOC and STN concentrations, and soil organic matter (SOM) concentration data from FD-based soil. The R script developed by von Haden et al. [61] was used to estimate ESM-based SOC and STN concentrations and stocks using cubic spline interpolation models. To acquire the SOC and STN stock down to 1 m depth, the SOC and STN stocks of the five soil layers were added up. Stratification ratio (SR) was calculated by dividing the SOC concentration of the surface depth layer (D1 layer) by those of the lower depth layers (D2, D3, D4, and D5). Thus, four SRs were defined: SR1 = D1/D2, SR2 = D1/D3, SR3 = D1/D4, and SR4 = D1/D5.

SOC and STN concentrations and BD_FS_ were derived from the same soil samples to allow accurate computation of SOC and STN stocks based on equivalent soil mass (ESM) as recommended by Ellert et al. [62] and Wendt and Hauser [63]. ESM-based SOC and STN stocks estimations may contain inaccuracies if separate samples are used for BD and SOC concentration [61]. Hereafter, ESM-based SOC concentration and stock are simply referred to as SOC concentration and SOC stock, respectively.

### Statistical analysis

The Welch´s t-test was employed to verify whether the parameters under study vary with land-use change, from MEF to BP fields. Wilcoxon test was also performed to determine whether the SR values were statistically ≥ 2, as SR values > 2 indicate higher soil quality and ratios < 2 are frequently found in degraded soil [64]. All statistical analyses were performed at α = 0.05, using R [65]. This investigation was carried out with the presumption that, before BP was established, the SOC and STN stocks of the soils presently under BP (which were under MEF at the time of its establishment) and the soils currently under MEF (the soils whose land cover did not change) were the same.

## Results

### Biomass and necromass pools

**Fig. 2:**
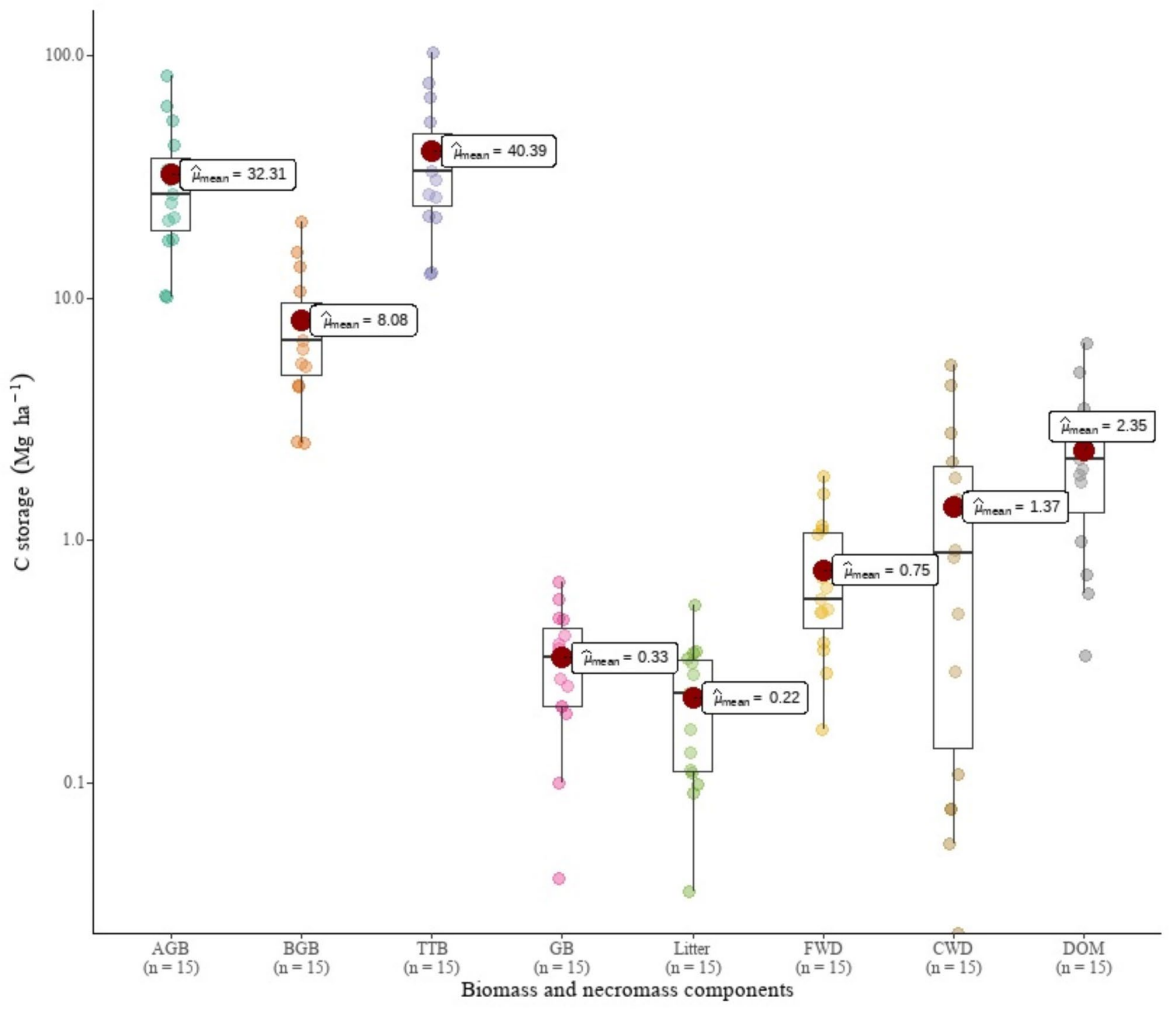
Carbon estimates in biomass and necromass pools of montane evergreen forest

There were no trees, grasses, tree litter, or CWD in BP fields. The C reserves in live tree biomass in MEF areas were estimated to be 40.39 Mg ha ^– 1^, with AGC accounting for 80% and BGC accounting for the remainder (Fig. 2). C reserves in dead organic matter (litter + FWD + CWD) were 2.25 Mg ha ^– 1^, with CWD accounting for 58% (Fig. 2). Overall, the conversion of MEF to BP resulted in a loss of biomass and necromass C stores of about 43.07 Mg ha ^– 1^.

### Soil pool

**Fig. 3:**
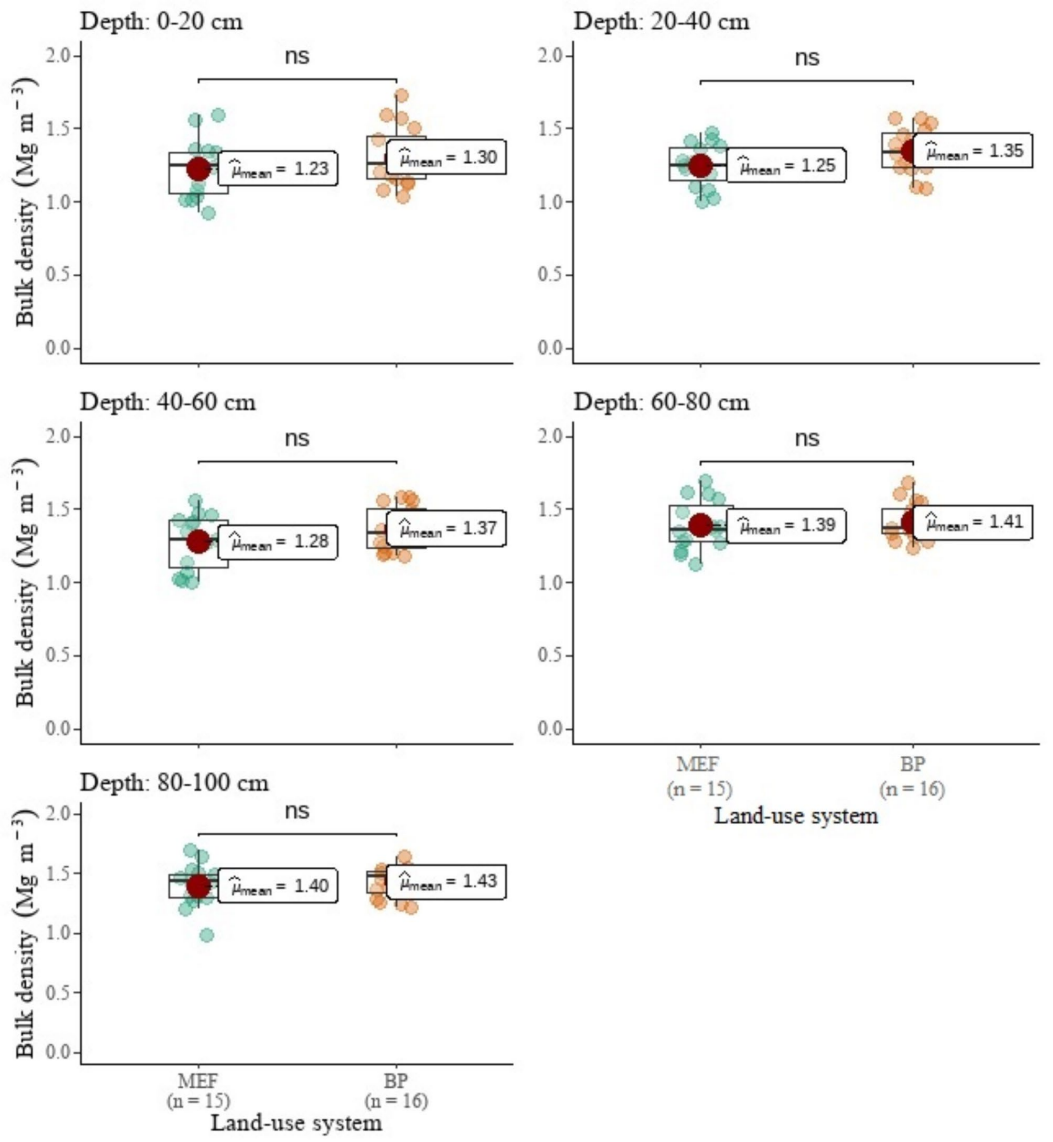
Comparison of land-use system means of soil bulk density. ns = not statistically significant. *, **, ***, **** = statistically significant at α = 0.05, 0.01, 0.001, 0.0001, respectively

**Fig. 4:**
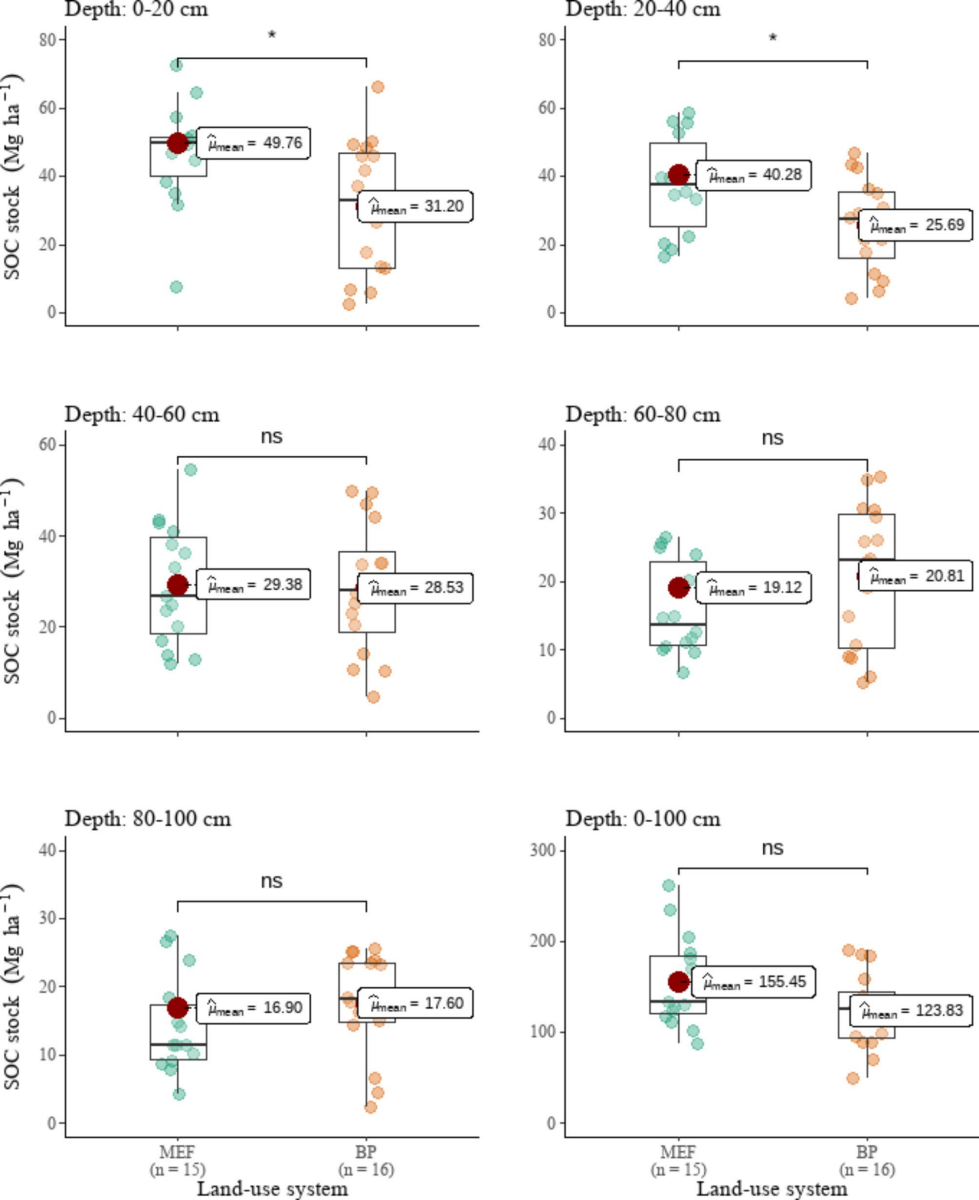
Comparison of land-use system means of soil organic carbon (SOC) stocks. ns = not statistically significant. *, **, ***, **** = statistically significant at α = 0.05, 0.01, 0.001, 0.0001, respectively

**Fig. 5:**
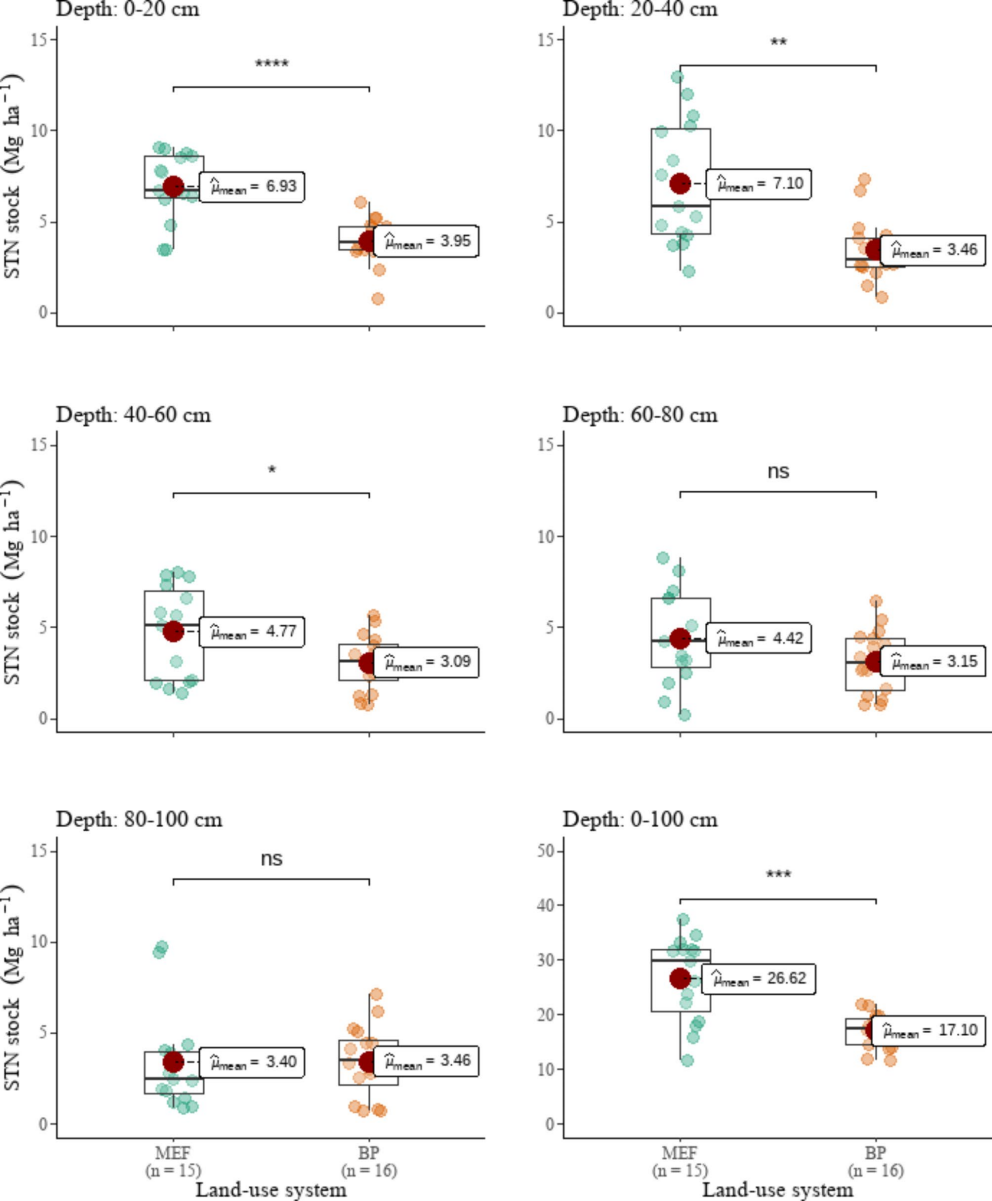
Comparison of land-use system means of soil total nitrogen (STN) stocks. ns = not statistically significant. *, **, ***, **** = statistically significant at α = 0.05, 0.01, 0.001, 0.0001, respectively

No statistically significant changes in BD were observed following MEF conversion to BP fields (Fig. 3). In terms of SOC stocks, MEF lands differed from BP fields only in the surface (D1: 0–20 cm) and subsurface soil (D1: 20–40 cm) (Fig. 4). However, they differed in total STN (Fig. 5) in the first three soil layers (D1, D2, and D3). SOC stocks of the surface and subsurface layers decreased by 37 and 36%, respectively, following MEF conversion to BP. When the whole 1-m soil depth was evaluated, SOC stocks did not differ significantly between MEF and BP fields. Nonetheless, the total SOC reserves in the BP fields (123.83 Mg ha ^– 1^) were 20% lower than in the MEF fields (155.45 Mg ha ^– 1^). The BP fields’ top three soil layers (D1, D2, and D3) were 43, 51, and 35% lower in STN than the MEF fields’, respectively (Fig. 5). The total STN to 1 m depth of MEF and BP fields were estimated to be 26.62 Mg ha ^– 1^ and 17.10 Mg ha ^– 1^, respectively, representing a 36% drop.

The cumulative SOC losses in the upper 40-cm depth layer recorded after MEF conversion to BF were calculated to be 37% (a fall from 90.04 to 56.89 Mg ha – 1). In contrast, MEF conversion to BF resulted in 44% cumulative STN losses in the upper 60 cm of soil depth.

**Fig. 6:**
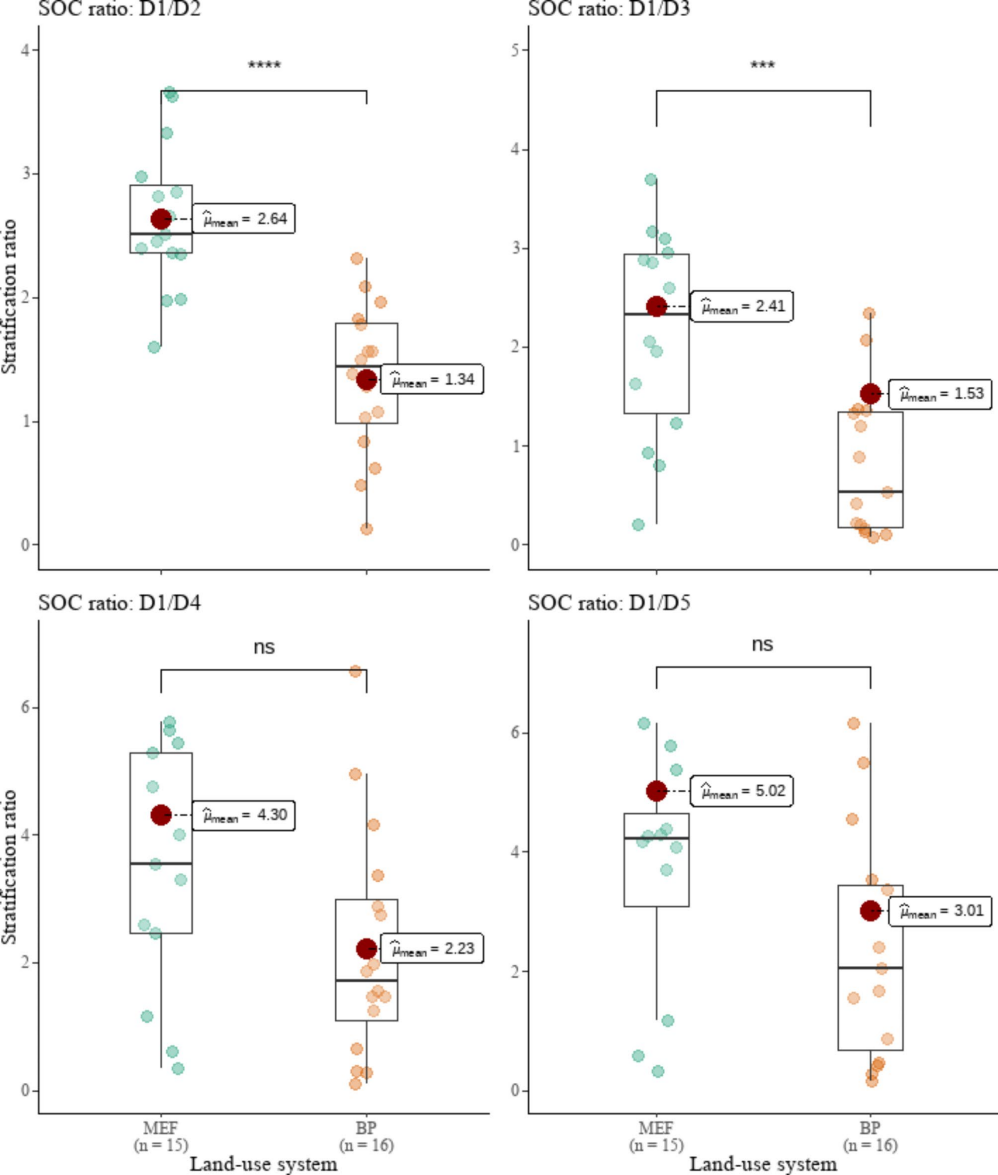
Comparison of land-use system means of stratification ratio (SR) stocks. ns = not statistically significant. *, **, ***, **** = statistically significant at α = 0.05, 0.01, 0.001, 0.0001, respectively

The SR for D1 to D2 and D1 to D3 dropped by 49 and 37%, respectively, following MEF conversion to BP (Fig. 6). All of the SR values of the MEF fields were found to be statistically greater than 2 (*P* = 0.001). The D1 to D2 and D1 to D3 SR of BP fields, on the other hand, were statistically inferior to 2 (*P* = 0.003). These findings imply that the quality of the topsoil degraded as MEF was converted to BP.

## Discussion

The current study’s estimates of the amount of C stored in the biomass (total tree C = 40.39, AGC = 32.31 Mg ha ^– 1^) are higher than estimates made for Mozambican dry *miombo* woodlands by Ribeiro et al. [66] (total tree C = 28.88 Mg ha ^– 1^), Woollen et al. (2012) (AGC = 20.7 Mg ha ^– 1^), Montfort et al. [25] (total tree C = 36.00 Mg ha ^– 1^), and Ryan et al. [67] (total tree C = 32.10, AGC = 21.20 Mg ha ^– 1^), but lower than those made for Mozambican mecrusse woodlands by Magalhães and Seifert [68] and Magalhães [69]( total tree C = 75.37 Mg ha ^– 1^, AGC = 60.56 Mg ha ^– 1^).

The current study’s relative SOC and STN stock losses on the topsoil after deforestation for agriculture are within the range of losses recorded for Mozambique. Following the conversion of woodlands to farmlands, Montfort et al. [25] showed a 43% decrease in SOC in the upper 30-cm, and Magalhães [22] found a decline of up to 72% in the topsoil (0–30 cm). The SOC and STN losses in this investigation, in contrast to the studies described above, extend to 40 and 60 cm depths, respectively. Additionally, with regard to the topsoil, the findings of this study are in line with those of sub-Saharan Africa. De Blécourt et al. [70] found agricultural lands to be 20–39% lower in SOC and STN in southern Africa. Touré et al. [71], Elberling et al. [72], Demessie et al. [73], Walker and Desanker [24], Hounkpatin et al. [74] reported SOC and STN losses ranging from 12 to 52% after deforestation for agricultural fields in Namibia, Senegal, Ethiopia, Malawi, and Burkina Faso. The current study also agrees with global studies and meta-analyses that reveal a decrease in SOC and STN stocks after agricultural deforestation [15–17, 75–77].

This study agrees with Powers [37], who discovered that converting forests to BP reduced SOC concentrations and stocks in the surface soil by 37% and 16.5%, respectively. In line with the findings of Biazin et al. [15], it was shown in this study that SOC stocks did not differ between MEF and BP fields when the entire 1-m soil depth was assessed. In this investigation, it was shown that the cumulative SOC and STN losses in the top 40 cm were more than the losses seen throughout the entire 1-m soil profile, which is similar to the conclusion by de Blécourt et al. [78].

Deforestation for BP had a decreasing effect on SOC and STN as soil depth increased: substantial variations in soil properties between MEF and BP fields were only seen in the top 40 cm for SOC and the top 60 cm for STN. This is consistent with the findings of Elberling et al. [72], Walker and Desanker [24], de Blécourt et al. [70], who found that the effects of deforestation for agriculture are frequently highest in topsoil. The topsoil is the most vulnerable to SOC and STN loss due to land-use changes [79–81]. For example, the topsoil of BP fields is directly disturbed by agricultural activities affecting negatively the soil properties; whereas, the topsoil of MEF lands receives direct nutrient return from leaf and root litter, impacting positively SOC and STN. This explains the differences in SOC and STN stocks in the topsoil of MEF and BP lands. The consequences of deforestation for BP fields were also more pronounced in the topsoil due to increased microbial activity and faster deposition rates there than in the subsoil [82], in addition to the aforementioned considerations.

Even though the majority of studies claim that SOC and STN changes resulting from land-use change primarily occur in the surface soil [15, 19, 83], in the upper 20–30 cm [16, 17, 84], SOC and STN losses in this study extended down to 40 and 60 cm, respectively. This agrees with the findings of Hounkpatin et al. [74] and de Blécourt et al. [70]. This could be due to topsoil and subsurface mixing during site preparation [38, 85].

The full removal of forest cover for BP disrupts or considerably reduces C inputs in the soil, resulting in an imbalance between C inputs and outputs, which explains the observed fall in SOC and STN stocks. Continuous cultivation with no supplemental input exacerbates the loss in SOC and STN [26, 86]. The primary cause of SOC and STN losses once forests are converted to agricultural fields is thought to be the massive export of nutrients by agricultural products [30]. However, agricultural products (banana) are not the exclusive source of nutrient exports in the research region. The use of banana pseudostems and leaves to build banana transportation baskets exacerbates nutrient export. This is compounded because no fertilizer is used to replenish the nutrients exported. The use of organic or inorganic fertilizers increases SOC and STN concentrations and stocks [87–91]. Furthermore, banana plants are reported to export relatively larger amounts of nutrients through fruit harvesting than other cultivated plants [27, 28], because of their greater nutrient demand [29].

Since the research region is situated in a mountainous environment with steep hillslopes, biological factors, such as fewer C inputs, are not the only explanation for SOC and STN losses. Physical removal by leaching and erosion [26], which are propelled by vegetation removal, may have had a substantial impact. Therefore, the following factors may have contributed further to SOC and STN losses: (1) the erodibility of the soils on the study area’s steep hillslopes, (2) the susceptibility of bare soils to erosion, and (3) the potential for BP to increase throughfall kinetic energy, throughfall volumes, and splash erosion through canopy dripping.

By catching rainwater, boosting infiltration, stabilizing soil aggregates, and lowering soil erodibility, vegetation protects soil surface from water and wind erosion [92–94]. As a result, the removal of vegetation cover increases soil erodibility, enhances landslide risk, and causes water and wind erosion, and, subsequently, causes losses in SOC and STN [95–98]. Furthermore, Ma et al. [99] and Alonso-Sarría et al. [100] showed that the soil erosion and loss of soil nutrients are increased when natural forests are converted to agricultural lands. According to Liu et al. [97], this phenomenon is notably exacerbated in steep hillslopes, which describes the study area in question, where the slope angle reached up to 42% (23^o^).

BP may have exacerbated rather than reduced the aforementioned consequences of forest clearance. When compared to open rainfall, the banana canopy is known to significantly redistribute atmospheric rainfall and to increase throughfall kinetic energy, throughfall volume, soil particle splash loss, throughfall erosivity, and splash erosion by up to five times [31–33].

There is general agreement that SOC and STN losses are influenced by the interaction between land-use changes, soil erosion, and, indirectly, banana canopy features. Therefore, additional research is required to determine how much each factor affects SOC and STN losses. Li et al. [34] concluded that SOC content in deep soil was mainly affected by factors related to land uses.

The results of the current study demonstrate that the conversion of MEF to BP fields resulted in a loss in soil quality, as SR dramatically reduced and eventually fell to levels below 2. Higher soil quality is indicated by SR values over 2, while ratios under 2 are frequently found in deteriorated soil [64, 101]. Continuous cultivations and mixing of topsoil and subsoil during site preparation could explain why SR values in BP fields are lower than in MEF lands. Deng et al. [102] found that SR is affected by the years following vegetation replacement and the years of farming.

## Conclusions

As a result of clearing MEF for BP, all carbon stores contained in live biomass and necromass were completely removed. The subsequent and ongoing cultivation of bananas on bare, steep hillslopes caused losses in SOC and STN not only in the top 20 cm of soil, but also in the subsoil, where losses in SOC were found to extend to 40 cm and losses in total STN to 60 cm. Cumulative loss down to 1-m depth was only significant for STN. The stratification ratio, a measure of soil quality recovery or deterioration following land use and land cover changes, indicated that the soil quality was depleted following deforestation for BP. Additional research is needed to validate the idea and assess the extent to which land-use change, soil erosion, and, indirectly, banana canopy dripping contribute to the aforementioned losses.

## Data Availability

Not applicable.
